# Adapting a Dementia Care Management Intervention for Regional Implementation: A Theory-Based Participatory Barrier Analysis

**DOI:** 10.3390/ijerph19095478

**Published:** 2022-04-30

**Authors:** Katja Seidel, Tina Quasdorf, Julia Haberstroh, Jochen René Thyrian

**Affiliations:** 1 Psychological Ageing Research, Department of Psychology, Faculty V: School of Life Sciences, University of Siegen, Adolf-Reichwein-Str. 2a, 57068 Siegen, Germany; julia.haberstroh@uni-siegen.de; 2 German Center for Neurodegenerative Diseases (DZNE), Site Witten, Stockumer Str. 12, 58453 Witten, Germany; tina.quasdorf@dzne.de; 3 Faculty of Health, Witten/Herdecke University, Alfred-Herrhausen-Straße 50, 58455 Witten, Germany; 4 German Center for Neurodegenerative Diseases (DZNE), Site Rostock/Greifswald, Ellernholzstr. 1/2, 17489 Greifswald, Germany; rene.thyrian@dzne.de; 5 Institute for Community Medicine, University Medicine Greifswald, Ellernholzstr. 1-2, 17489 Greifswald, Germany

**Keywords:** dementia care, care management, participatory research, barrier analysis, implementation research, consolidated framework for implementation research

## Abstract

Dementia is a leading cause of disability and dependency in older people worldwide. As the number of people affected increases, so does the need for innovative care models. Dementia care management (DCM) is an empirically validated approach for improving the care and quality of life for people with dementia (PwD) and caregivers. The aim of this study is to investigate the influencing factors and critical pathways for the implementation of a regionally adapted DCM standard in the existing primary care structures in the German region of Siegen-Wittgenstein (SW). Utilizing participatory research methods, five local health care experts as co-researchers conducted *N* = 13 semi-structured interviews with 22 local professionals and one caregiver as peer reviewers. Data collection and analysis were based on the Consolidated Framework for Implementation Research (CFIR). Our results show that among the most mentioned influencing factors, three CFIR constructs can be identified as both barriers and facilitators: Patients’ needs and resources, Relative advantage, and Cosmopolitanism. The insufficient involvement of relevant stakeholders is the major barrier and the comprehensive consideration of patient needs through dementia care managers is the strongest facilitating factor. The study underlines the vital role of barrier analysis in site-specific DCM implementation.

## 1. Introduction

The increasing numbers of people with dementia has been identified as a challenge for health care systems worldwide [1]. To tackle this challenge, governments have issued dementia strategies, that target fields of action. In Germany, the comprehensive *National Dementia Strategy* (NDS) was adopted in September 2020. One overarching goal was increasing action in the development and transfer of evidence-based prevention and care concepts into routine care [2]. The NDS includes dementia care management (DCM), a specific measurement to overcome a variety of challenges in routine care. DCM is a collaborative model of care. By integrating multimodal and multiprofessional strategies, the aim is to provide individualized and optimized care within the framework of the established health care system [3,4]. The DCM standard comprises three major topics, the so-called *pillars*: management of treatment and care, medication management, and caregiver support and education [5]. These three pillars comprise eight different *action fields* and, among them, a total of 25 *foci*, which include a total of 95 specific DCM interventions [5] (p. 251). All of them target the needs of people with dementia (PwD) and their dependents. The centerpieces of DCM are dementia care managers, qualified care professionals who systematically (with computer support) assess the complex medical, psychosocial, and care needs of PwD and family caregivers in the home setting. Based on these assessments, they draft an individual care program consisting of DCM interventions and continuously support and monitor its realization. Safety, efficacy, acceptability, and cost-effectiveness have been demonstrated in the cluster-randomized controlled intervention trial *DelpHi-MV* (“Dementia: life- and person-centered help in Mecklenburg-Western Pomerania”), and the intervention has been described in great detail [3,4,5,6,7]. However, for various reasons, this evidence-based concept has not been transferred into routine care in Germany. The process of implementation has now been initiated by the NDS [8]. One success factor for the local implementation of particularly complex, evidence-based, multilevel health care interventions such as DCM is involving relevant local stakeholders by using participatory methods [8,9]. Such an approach allows the inclusion of local knowledge, insights, experiences, perspectives, concerns, and priorities and can increase the likelihood of successful implementation [9]. Integrating target group-specific expertise through participation improves the research process and increases the likelihood of a needs-based intervention that also considers context-specific factors [9,10,11].

**Figure 1:**
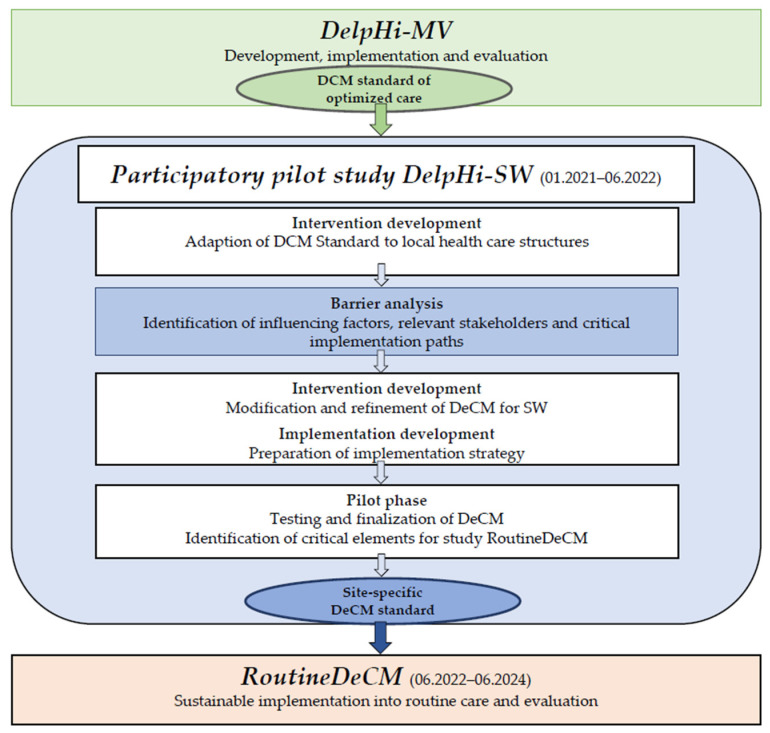
Embedding of the barrier analysis.

The study presented here is part of the *Participatory pilot study DelpHi-SW* (Dementia: life- and person-centered help in Siegen-Wittgenstein). DelpHi-SW aims to test a structured participatory approach to adapting and implementing the original DCM standard to an exemplary local setting (Siegen-Wittgenstein: SW). As depicted in Figure 1, the first step of DelpHi-SW was to adapt the DCM standard to the local health care structures. In the adapted version, the DCM standard is abbreviated as DeCM.

The second step is a barrier analysis. Based on the results of the barrier analysis, the adapted DeCM standard will be refined again. It will then be tested in a pilot phase based on a small sample size in SW and adjusted, if necessary, in an iterative adaptation process. Finally, an implementable site-specific DeCM standard will be manualized and evaluated in a subsequent study, *RoutineDeCM*, regarding its ability to be sustainably implemented in routine care. This approach will be transferable to and replicable in other local settings to guide the implementation processes of DCM in different local settings. 

In this article, we present the results of the barrier analysis because, on the path to sustainable implementation, it is highly relevant to consider factors that may hinder implementation efforts. Ideally, this is done using a framework model that guides and thereby promotes implementation success already in the stage of pre-implementation [12,13]. The aim of the barrier analysis is to identify intervention- and setting-specific key factors that potentially influence the implementation of DCM in routine care. This analysis integrates the identification and consideration of regional resources and specificities as well as factors that may hinder the implementation efforts within the adaptation process.

## 2. Materials and Methods

### 2.1. Design

The analysis of barriers follows a qualitative design using semi-structured interviews. The reporting of the methods applied in this study is aligned with the Consolidated Criteria for Reporting Qualitative Research (COREQ) [14] and the Standards for Reporting Qualitative Research (SRQR) [15]. The SRQR checklist is provided as a supplement (Table S1). 

### 2.2. Participatory Approach

Five local healthcare experts (four female) from four different healthcare sectors (an Alzheimer’s society, a local medical network, a care and welfare association, and a hospital) were involved as co-researchers. They were trained dementia care managers in participating institutions with established care structures, and they were familiar with DCM. The co-researchers interviewed other regional stakeholders as peer reviewers. 

### 2.3. Theoretical Framework

The Consolidated Framework for Implementation Research (CFIR) is an actionable framework model that guides the data collection, coding process, analysis, and interpretation of findings in this study [13]. The CFIR comprises 39 constructs that are associated with effective implementation. The constructs are assigned to five major domains and refer to the characteristics of (1) intervention, (2) outer and (3) inner setting, (4) individuals, and (5) process. These domains with subsumed constructs influence the effectiveness of implementation activities and are interrelated in complex ways [13]. (1) Characteristics of the intervention include all the basic and indispensable elements of an intervention, but also those components that may still be modified [13]. Inner and outer settings refer to the contexts of an organization in which an intervention will be implemented. (2) Outer setting refers to contexts outside the implementing organization (e.g., social, political, and legal factors). In comparison, (3) inner setting refers to all features within the organization that might affect and get affected by the implementation (e.g., structural characteristics, culture, climate, and networks) [13]. (4) The characteristics of individuals refer to features of persons directly or indirectly involved in the implementation process or intervention (e.g., knowledge, beliefs, and personal attributes) [13]. All activities during the implementation process are subsumed under (5) characteristics of the process (e.g., planning, engaging) [13]. This meta-theoretical framework was chosen as it has been proven effective in systematically identifying the key inhibiting and facilitating factors as well as actionable information for the successful implementation of complex, multilevel health care interventions such as the DCM standard [12,13,16,17]. Furthermore, its standardized structure fosters comparability of the results of different evaluations across the different phases of the DeCM implementation process. At the same time, CFIR enables and encourages individual adaptation to specific requirements of different implementation settings or to specific issues throughout different stages of implementation processes [16]. 

### 2.4. Sampling

The peer reviewers were selected using purposive sample strategies [18] based on critical discussions during the adaptation phase of the DCM standard. All peer reviewers (*N* = 23, 17 female) were recruited by the co-researchers from their networks in SW. As the function and responsibility of doctors (*n* = 2), nurses, and care service providers (*n* = 7), palliative care providers (*n* = 1), regional networks (*n* = 1), hospitals (*n* = 4), counseling services (*n* = 4), and self-help services (*n* = 3) in the adapted DeCM standard were discussed. As these stakeholders are highly relevant in the provision of dementia care in SW, this target group was chosen. Additionally, one caring relative was interviewed to obtain critical feedback outside the professional care network. The peer reviewers represent the different functional and hierarchical levels of the institutions to obtain as many user perspectives as possible. Moreover, peer reviewers represent the possible user group of DCM regarding the gender aspect since approximately 75% of the employees in health care professions in Germany are female [19]. 

### 2.5. Material

The interview guide (Supplementary Materials File S1) was developed based on the CFIR and the associated online interview guide tool [20]. CFIR constructs relevant for the interview guide were identified based on the study results thus far and were discussed with the research team. The questions were then adapted to the context of the study (e.g., “What difficulties could arise in the implementation of the new form of care for PWD and their relatives here in SW?” [patient needs & resources]; “Who needs to be engaged in SW to implement DeCM in our region?”, “Who are the people/institutions that can drive the successful implementation of DeCM?” [engagement]) and accompanied by brief explanations for the co-researchers about the underlying constructs. Additionally, a manual (including information on the background, methodology, and procedure) was provided for preparation, and the co-researchers were offered individual preparatory consultations by an academic researcher. The specific aspects of the adapted DeCM standard (pillars, action fields, foci, and interventions) to be discussed were selected by the co-researchers. The focus was on aspects (1) that were critically discussed during their conception, (2) that had been modified or newly developed during intervention development compared to the original DCM standard, (3) that were more relevant for the organization’s own work setting, and (4) where additional expertise and input were needed. Since the adapted DeCM standard was available only as an abstract working model, the co-researchers accepted the proposal that the academic research team transforms the content aspects into text vignettes. 

### 2.6. Data Collection

The barrier analysis was conducted using semi-structured interviews between July and November 2021. Of a total of 13 interviews, 12 were organized, prepared, and conducted by the co-researchers. One interview was conducted by two academic researchers (Katja Seidel, Jochen René Thyrian) because the co-researchers were too involved in their regular work in inpatient care. All interviews took place in the work context as face-to-face interviews or digital interviews (*n* = 2). The interviewees participated voluntarily in interviews alone or in groups (max *n* =5). The interviews took between 45 min and 90 min. Two interviews were recorded with the permission of the peer reviewers, as they were group interviews. Afterward, the records were transformed into summary protocols. For the other interviews, to allow the experts to concentrate on the interview, one of two research interns (trained for the task) wrote a summary protocol with quotes and paraphrased and summarized statements. Key statements were repeatedly reported to the interviewees during the interview and corrected for incorrectly or misleadingly recorded content if necessary to check the accuracy. To be able to assess the significance of inhibiting and facilitating aspects, the co-researchers were asked to rate the following statements: “On a scale of 1 (not at all relevant) to 4 (highly relevant), how relevant do you consider this aspect to be?”. In addition, the characteristics of the interviews, interviewees, and field notes were documented in an observer guide (Supplementary Materials File S2) by the research interns. It was up to the experts to decide when no more incremental information could be obtained and the interview could be terminated.

### 2.7. Data Analysis

The data were analyzed using qualitative content analysis [21] based on the deductive application of the CFIR constructs [13]. At the first level of analysis, one academic researcher (Katja Seidel) assigned all individual text units from all interviews to the CFIR domain and categories according to the CFIR coding criteria [22]. A second researcher independently coded the text units of five randomly chosen interviews using the same CFIR coding scheme. As the intercoder reliability (*rH* = 0.70) was below the recommended threshold [23], a third researcher (Tina Quasdorf) reviewed the results. The data material was discussed in terms of the inconsistent units of analysis. The coding guidelines for the data material under review were refined, and the data were thus analyzed again in a second run. In the second step, the assigned text units were then condensed in terms of their central statement, and meaning units were generated. After the entire data coding process was completed, the data were randomly reviewed again by an experienced researcher.

## 3. Results

A total of 516 text units were coded to 27 CFIR constructs within all CFIR domains. Twelve of the CFIR constructs were not applied in the coding. Therefore, 40 text units (7.8%) were assigned to more than one CFIR construct due to their content-related statement. After condensing all text units, 418 codings remained, over half of which were assigned to barriers to implementing the adapted DeCM standard (59.6%). Table S2 presents the overall results of the coding process. The statements of the peer-reviewers regarding barriers to the regional implementation of DeCM were most frequently coded to the CFIR constructs *Engaging* (24.5% including according subconstructs; CFIR-domain *Process*), *Patient Needs and Resources* (14.9%; CFIR domain *Outer Setting*), *Relative Advantage* and *Trialability* (both 10%; CFIR domain *Intervention Characteristics*), and *Cosmopolitanism* (8.8%; CFIR domain *Outer Setting*). The statements of the interviewees regarding facilitators of the regional implementation of DeCM were most frequently coded to the CFIR constructs *Patient Needs and Resources* (24.9%; CFIR domain *Outer Setting*), *Relative Advantage* (20.1%; CFIR domain *Intervention Characteristics*), *Implementation Climate* (19.5%; CFIR domain *Inner Setting*), *Readiness for Implementation* (8.3%; CFIR domain *Inner Setting*), and *Cosmopolitanism* (5.9%; CFIR domain *Outer Setting*). Three CFIR constructs represent both barriers and facilitators and are therefore presented together in the following section. The following results, organized by CFIR constructs, focus on the barriers and facilitators that most jeopardize or promote the implementation of DeCM in SW. 

### 3.1. CFIR Constructs Relevant as Barriers and Facilitators

#### 3.1.1. Patient Needs and Resources (CFIR Domain Outer Setting)

This construct refers to the extent to which the adapted DeCM intervention addresses patient needs [13]. The present results show that the anticipated barriers within this construct mostly relate to specific DeCM interventions. For example, the implementation of a dementia screening for all persons over 65 years as a standard of a routine basic assessment by General practitioners (GPs) and during inpatient hospitalization was conceptualized. This was sharply criticized by most reviewers and was considered disproportionate and “incapacitating” (Interview 11).

“Why do we disenfranchise people with dementia to make their own decisions, but not with any other disease? The measure assumes that every person with dementia wants to have action taken. Screening is and should always be voluntary, also with dementia.”

(Interview 11)

“Screening of all patients over 65 is a joke, because working until 70 and becoming screened is a social problem.”

(Interview 9)

A doctor’s consultation raising awareness of the disease with voluntary diagnostics, creating suitable test conditions, and providing further counseling services was suggested as a procedure that would take patients’ needs into account. Additionally, the counseling services developed in the DeCM standard within the framework of diagnosis were criticized because a large amount of information would overburden PwD. There were also criticisms that the DeCM standard focuses only on the main caregiver and does not take the entire family system into account. Furthermore, there was skepticism because the acceptance and willingness to make use of DeCM depends significantly on the patient’s acceptance and motivation as well as the patient’s acceptance of the diagnosis. In this context, one expert referred to an observation in the counseling setting of an educational effect in which the willingness to use counseling services decreases with a higher level of education.

“The industrialists do not come to the counselling centers…the more upper class may not be picked up.”

(Interview 10)

There was also criticism of the lack of (a) consideration of PwD with a migration background and the corresponding linguistic barriers, (b) the topic of spirituality, and (c) consideration of younger PwD. One concern that was rated as highly relevant is that DeCM is not a low-threshold, easy-to-explain program. To make the concept understandable for people with dementia, a linguistic adaptation would have to be made.

Nevertheless, the results also show that many components of the adapted DeCM standard consider the needs of PwDs and primary caregivers and meet the increasing demand for home-based care. The support conceptualized in DeCM for primary caregivers, e.g., by recommending specific support services, is perceived as a necessary and important component of future dementia care, which can also strengthen the resilience of relatives and provide confidence. In particular, the recommendation of self-help groups by GPs already at the stage of diagnosis and as a supplement to medical counseling would consider issues such as the fears, excessive demands, and insecurity of caregivers following diagnosis.

“It is right to provide information at such an early stage. Relatives are probably overwhelmed, have uncertainties and fears that are addressed by the self-help groups. They are important...and another source of information than just medical counselling.”

(Interview 8)

This would be a solution to the problem of GPs’ limited time and resources in providing care. It is strongly assumed that the conceptualized information set in DeCM on the impact of the course of the disease on the communication skills of PwD will also strengthen the communication skills of relatives.

“Training and information on how the nonverbal and verbal communication of the PwD changes due to the disease helps relatives to be able to communicate with PwD.”

(Interview 6)

Furthermore, the designed actions in the DeCM standard on the topics of support for relatives, social integration, and strengthening the mental health of caregivers are highly appreciated. In general, the comprehensive consideration of counseling and support services is evaluated as highly appropriate. An advantage is seen, for example, in the fact that dementia care managers direct relatives to the counseling possibilities of self-help groups that address needs that are not covered by medical counseling. According to the reviewers, comprehensively designed dementia diagnostics can have a beneficial effect on building trust between PwD and professional stakeholders.

“But the complex diagnosis would have a beneficial influence on the relationship. Diagnosis would be time-consuming and lengthy. This allows PwD to get to know the nurses and doctors better and to build trust.”

(Interview 3, summarized field note)

The planned screening for dementia when it is suspected during a hospital stay is also considered very useful and reasonable by some peer reviewers.

“Even if patients are admitted with somatic symptoms but there is a suspicion of dementia, they should be screened for dementia.”

(Interview 7, summarized field note)

The strongest facilitating factor, according to the reviewers, is the use of dementia care managers. These professionals are seen as extremely beneficial. They are seen as important sources of support for PwD and caregivers, “bridging gaps in care” (Interview 8), and providing meaningful support in the orientation, coordination, and utilization of services in the existing health care system.

“PwDs increasingly want home care, and relatives are looking for ‘a framework’ of support. This is what dementia care managers offer.”

(Interview 5, summarized field note with direct quote)

“The discontinuities in care, that is, after the diagnosis by the GP it usually stops, are addressed by the dementia care managers.”

(Interview 8, original summarized field note)

In their function as permanent and coordinating contact people, dementia care managers are the link between care recipients and GPs.

“If the GP provides a person [dementia care manager] who ‘holds all the strings and knits with them’, this is useful.”

(Interview 5, original summarized field note with direct quote)

Reducing the number of contact persons in PwD care to one dementia care manager could give patients a sense of certainty, ease the burden through support, and reduce the fear of dementia. These professionals’ objective perspective would also make a valuable contribution to monitoring the course of the disease, and their home visits would enable a more comprehensive view of PwD.

“The operational blindness of relatives can lead to relatives not discovering emerging problems and symptoms. Dementia care manager would have a more independent perspective. When dementia care managers make home visits, the PwD is therefore seen in a more profound way.”

(Interview 4, summarized field note)

In addition, these managers are perceived as guides who can provide help tailored to individual needs. Their involvement is also assessed as beneficial, as they could ensure the transition from the inpatient to the outpatient setting and the connection supply in the latter. A standardized procedure for the transition to home care, as conceived in DeCM, is considered advantageous since, in some cases, no adequate follow-up care exists to date. The planned procedure for discharge and transition management would thus meet the needs of PwD and relatives to a high degree and would solve existing challenges in the care system in SW.

#### 3.1.2. Relative Advantage (CFIR Domain Intervention Characteristics)

This construct includes the perception that the implementation of an intervention is associated with benefits over an alternative or no change at all [13]. A large majority of reviewers perceive DeCM as a comprehensive model of care that goes beyond the current status quo.

“That is *the* [emphasis added] concept. There needs to be a central person who pulls all the strings, who coordinates, who supports people according to their needs. If we want PwD to continue to live at home and to participate in social life, it is the most workable concept.”

(Interview 11)

“That it goes beyond patient goes to neurologist, gets prescribed medication, is sent home.”

(Interview 1, summarized field note)

In their assessment, the deployment of dementia care managers plays a major role in offering better and more useful care than care as usual. DeCM would offer the possibility of overcoming the existing sectoring in the German health care system through dementia care managers as a central and coordinating point for the care of PwD. This mainly concerns the handling of discharge management in the inpatient setting.

“This is what will help people.”

(Interview 10)

On the other hand, the reviewers mentioned that an advantage of dementia care managers over the current care structure only exists if their responsibilities include the coordination of cross-sectoral services and not the provision of services already available in the SW. However, the reviewers also voiced objections that the adapted DeCM entails additional work for GPs and therefore does not represent an advantage over status quo care. This additional workload would be mainly due to an increased communication effort through the exchange with dementia care managers.

“Would be only advantageous if dementia care managers do their job well (work out a solid fundament with patients they can work with, not call GPs as soon as they encounter a small problem so GPs have to solve everything) otherwise it will be additional work.”

(Interview 1, summarized field note).

However, some reviewers offer contrary assessments, stating that the workload for GPs would be reduced as “DeCM is precisely responding to the lack of time of GPs”. (Interview 8). The reference that DeCM is not a low-threshold program that is easy to explain also leads to a critical questioning of the relative advantage of DeCM, which is why this aspect is also considered at this point.

At the level of concrete interventions, the reviewers see advantages and disadvantages over the existing approach. In particular, the planned implementation of dementia screening as a standard of routine assessments in GP practices and hospital admissions is criticized. Some experts describe the planned approach as “dictatorial” (Interview 11), “incapacitating” (Interview 11), and “too general” (Interview 7). Furthermore, some details of the planned dementia-specific medication management in the adapted DeCM standard are assessed as irrelevant or redundant. For example, the planned implementation of a drug check by pharmacies for side effects of anti-dementia drugs prescribed by neurologists is not seen as necessary. This is because this would already be standard and could lead to unnecessary uncertainty among people affected. However, some reviewers assessed the planned activities for the diagnosis of dementia as more comprehensive than the status quo.

“The new approach [recognizing dementia in time] would improve coverage of grey areas in diagnostics), diagnostics is facilitated.”

(Interview 3, summarized field note)

Furthermore, the central role of GPs as the primary point of contact for those affected within the framework of DeCM is perceived as advantageous. The planned interprofessional case discussions could even reduce the workload for the professionals involved. Further counseling of patients after diagnosis offered by dementia care managers and self-help groups would also represent a meaningful delegation of tasks. The planned DeCM interventions for comprehensive information of relevant actors in SW could lead to improved early detection of affected citizens in the future.

“It was discussed that comprehensive education about dementia in the whole region can lead to early detection of dementia, because postal workers, cashiers, etc. can also contribute to this.”

(Interview 3, summarized field note)

The fact that GPs should already refer to self-help services at the stage of diagnosis is perceived as new and sensible.

“The idea of offering self-help directly at diagnosis is considered a novelty.”

(Interview 8, summarized field note)

#### 3.1.3. Cosmopolitanism (CFIR Domain Outer Setting)

This construct describes the degree of connectedness of an organization with external organizations [13]. In our analysis, we have subsumed all statements that refer to intersectoral networking and communication between health care providers. According to the results, there is a high degree of skepticism and a highly rated need for improvement in the cooperation between the different care settings in SW to successfully implement the adapted DeCM standard. This relates to a lack of communication and cooperation at the sector boundaries, especially between hospitals, medical specialists, and GPs.

“Information about medication is only partially communicated, GPs and nurses communicate with the hospital, but the hospital hardly communicates with the nurses and the GP; one-way communication that works very difficult; It is important that communication works at institutional boundaries and that the individual sectors communicate with each other.”

(Interview 2, summarized field note)

“Difficulties are seen mainly in the lack of communication between each other.”

(Interview 5, summarized field note)

“Difficult points are where the sectors must collaborate.”

(Interview 11)

“Over time, all healthcare sectors have become lone warriors.”

(Interview 5)

According to the reviewers, communication and collaboration would succeed only through the commitment of nursing staff. Furthermore, the telematic infrastructure necessary for successful implementation would not be available to all project participants. There would be a high demand for linking all sectors through a common digital platform within the framework of discharge and transfer management.

“Urgent need for intersectoral cooperation, especially in the area of discharge and transfer management.”

(Interview 8, summarized field note)

In this context, communication from hospitals to doctors’ practices is inadequate, which hinders the follow-up treatment of PwD with medication. In addition, hierarchical structures and competition hinder intersectoral communication and cooperation between nurses and doctors. However, features of the pilot study itself were mentioned in the interviews as a facilitating factor for implementing the adapted DeCM standard. Accordingly, the participatory, cross-sectoral approach of the project would allow a high degree of networking, which is considered essential for implementation.

“’It is a collaborative project that involves many sectors. It is good that it is participatory.’ (On request: The participatory approach is a facilitating factor)”

(Interview 2, summarized field note with direct quote)

“A high degree of collaboration is necessary for the implementation of DeCM, but this is given in the pilot study and is seen as an advantage. Use of the *Gesundheitsregion Siegerland* as a project partner could solve the problem of missing referrals by GPs after diagnosis.”

(Interview 7, summarized field note)

There are regional network structures through which many actors are already linked with each other or want to become involved in the future, which is assessed as a supportive factor for regional implementation.

“However, a concept for network structures was developed in cooperation with the University of Siegen and the SW region. The institution will be more involved in this framework from next year onwards.”

(Interview 13, summarized field note)

Additionally, the digitalization in all sectors driven by the COVID-19 pandemic would be an opportunity for the implementation and support of connectivity between sectors.

“Digitalization had progressed differently in the different sectors. Corona has triggered digitalization, which could be beneficial for discharge and transfer management.”

(Interview 2, summarized field note)

The cross-sectoral exchange would also be promoted and supported by telematics infrastructure. Here, the use of electronic patient files will be particularly important. There are regional network structures through which many actors are already linked with each other or want to become involved in the future, which is assessed as a facilitating factor.

### 3.2. CFIR Constructs That Are Exclusively Barriers

#### 3.2.1. Engaging (CFIR Domain Process)

This construct, with its subconstructs, includes the degree of involvement of relevant stakeholders for successful implementation. These include people who are formally responsible for implementation (implementation leaders), support implementation (champions), influence the mindsets of others (opinion leaders), or formally make intervention decisions as external actors [13]. All interviewees identified regional stakeholders who (a) are highly relevant for the successful implementation process in SW or (b) are not yet considered in the adapted DeCM standard itself. The statements were coded under barriers, as they indicate an open need for action that would jeopardize implementation if not considered. GPs who are not part of the study-specific cooperation agreement were identified as central but missing actors who should know about and promote DeCM as a central point of contact for patients. Outpatient and inpatient neurologists, other specialists as well as regional hospitals, and specifically geriatric units were also mentioned as relevant stakeholders. The inclusion of these stakeholders could improve the intersectoral care of PwD. Specifically, it was emphasized that the responsibility for creating appropriate conditions for the implementation of time-intensive but highly relevant discharge and transition management in the adapted DeCM standard lies at the management level: “Discharge and transition management is important and if there is little time for it, then the management needs to allocate more time. This must be addressed to the management.” (Interview 2, summarized field note).

Furthermore, it must be ensured that the nursing staff are included in further implementation efforts, as they are specifically responsible for the implementation of discharge and transfer management in practice.

“Are the nursing staff of the hospitals involved? They have to conduct the discharge management and the transition; they must be actively involved.”

(Interview 2, summarized field note)

It was often pointed out that municipal institutions are both a resource in the DeCM standard and highly relevant for the implementation itself in SW. Social services, the care authority, the social welfare office, and the district care authority were repeatedly mentioned in this context. In addition to the overlaps, however, individual topics were specifically mentioned. For example, for the still incompletely conceived topic area of palliative care, the reviewers identified very specific contact persons and networks to be considered in SW, such as outpatient hospice services and palliative physicians. A newly identified and still incompletely designed part in the site-specific DeCM standard aims at the early detection of dementia. From the point of view of the reviewers, the inclusion and sensitization of banks, ambulance staff, hairdressers, and the general population is necessary to identify affected persons in everyday life well before diagnosis. Overall, the results show that the inclusion of further regional offers, networks, stakeholders, and institution-specific campaigns is a critical factor for successful implementation.

#### 3.2.2. Trialability (CFIR Domain Intervention Characteristics)

This construct refers to whether an intervention can be tested on a small scale [13]. In the analysis of the results, we have subsumed statements that contain reasons for the non-trialability of the adapted DeCM standard. GPs play a central role in the care of PwD within the framework of DeCM. The peer reviewers note that an existing overload of GP practices and limited time capacities hinder practical implementation in care practice. Furthermore, the high implementation costs in hospitals should be viewed critically. Specifically, the fact that highly qualified nursing staff are missing in the day-to-day business of the wards due to their role as dementia care managers can hinder trialability in the hospital setting. Overall, the reviewers repeatedly note that the complexity of the DeCM standard goes hand in hand with a “high time expenditure for implementation” (Interview 7, summarized field note) and represents a considerable barrier.

“(...)complicated (...) DeCM also, because many sectors must be involved and get on board. Complicated, but solvable, because some sectors are interested in it, and it also relieves workload for doctors.”

(Interview 11)

Furthermore, some reviewers assume that regional implementation depends to a large extent on the sufficient motivation of all participants, the degree of conviction of the GPs, and cooperation at the intersectoral care interfaces in SW. According to the reviewers, the complexity of the DeCM standard increases the coordination effort, and despite the perceived usefulness, its implementation would be a lengthy and challenging process. Further critical aspects concern attitude and behavior. For example, the experts perceive that implementation is accompanied by a change in previously common care processes as well as in the competency and responsibility structures in the care of PwD. In this respect, they question whether an expansion of the responsibility of dementia care managers and a possible shift in professional competencies and tasks will be supported by all physicians. In this context, the lack of willingness to accept change on the part of nursing staff could also hinder practical implementation.

### 3.3. CFIR Constructs Relevant Exclusively as Facilitators

#### 3.3.1. Implementation Climate (CFIR Domain Inner Setting)

This construct refers to the readiness within an organization to implement change, the extent to which stakeholders embrace a new intervention, and the extent to which the implementation of this intervention is encouraged, endorsed, and expected within the organization [13]. It relates to the need for change in the status quo (the tension for change) and whether there is a fit between an organization and a new intervention (compatibility). Overall, all reviewers agree that there is a strong need for DeCM.

“There is a great need, especially because many PwD and their relatives do not yet know about dementia and its implications for the care of PwD at the beginning of the disease.”

(Interview 4, summarized field note)

The demographic development in SW and the increasing number of people affected by dementia argue in favor of implementation, and the reviewers feel that their sectors are ready for it. Some signal their willingness to employ dementia care managers, as “It would not be seen as a big change, but only as a relief” (Interview 5, summarized field note). 

Several evaluators emphasize the fit between the range of their tasks within the adapted DeCM standard and the planned internal orientation of services and offers for PwDs.

“It ‘fits well’, basically the processes have to be adapted to PwD anyway. Dementia is a big issue for the facility, they care for many PwD.”

(Interview 5, summarized field note with direct quote)

Several reviewers expressed their willingness to engage in further dialogue and referred to their existing networks. They stated that they see a congruence and compatibility between the goals and processes in DeCM and their own internal structures and services, perceiving that this would facilitate its successful integration into existing internal structures and workflows.

“For the institution, a new reconciliation form is not a ‘new procedure’, but one that was already tried to be established before DelpHi-SW.”

(Interview 2, summarized field note with direct quote)

This is especially true in counseling services. This would be supported by existing established infrastructures, sufficient qualification, experience in the field of care and case management, and specifically the use of assessments.

“Many assessments are already conducted at the institution, and it has care and case management, which is why the changeover is not so significant.”

(Interview 3, summarized field note)

The availability of motivated and flexible stakeholders is considered a facilitating factor for implementation. Specifically, for discharge and transfer management, reviewers refer to existing established structures as well as existing standards on dementia in the inpatient setting. Here, for example, dementia officers are already employed, and the activities of dementia care managers are seen as complementary. Specifically, they would improve discharge and transfer management, and relevant actors would welcome dementia care managers. 

#### 3.3.2. Readiness for Implementation (CFIR Domain Inner Setting)

This construct includes statements about the extent to which an organization is ready to implement the innovation. In our analysis, this readiness refers to the commitment of managers, the availability of resources for implementation, and access to knowledge and information on the intervention (as subconstructs). As representatives of their settings, reviewers consider themselves suitable partners for the implementation of counseling services within the adapted DeCM standard, and some express the desire and interest to be further involved in the implementation process.

“Institution thinks it should be involved.”

(Interview 7, summarized field note)

Some reviewers also signaled their willingness to employ dementia care managers.

“(…) ’Hiring a DeCM? Yes, absolutely.’ It is considered a great asset.”

(Interview 5, summarized field note with direct quote)

In the view of several reviewers, there are sufficient internal organizational resources such as facilities, educational offers, access to technical infrastructure, existing electronic processing systems, and qualifications to implement DeCM in their structures.

The key aspects reported in the results section are summarized in Table 1, sorted by the five most important CFIR constructs.

## 4. Discussion

Our study showed that many barriers but also a considerable number of facilitating circumstances for implementing the adapted DeCM standard in routine care exist. Several of the individual aspects identified for implementing the DeCM standard can also be found almost 1:1 as barriers or facilitators in other studies in the field of dementia care services. This concerns, for example, the lack of information on service provision as a structural barrier to post-diagnostic care for PwD, the possible unwillingness of persons living with dementia to accept assistance, the lack of service suitability and patient-centeredness of services, and the positive assessment of care managers due to their networking and navigating function [24]. Based on the results, it was possible to identify among the multitude of potential influencing factors described by the CFIR those that appear particularly relevant for implementation in the region. There are three constructs that are relevant as both potential barriers and facilitators to implementation: *Patients needs and resources*, *Relative advantage*, and *Cosmopolitanism*. Furthermore, the *Engaging* and *Trialability constructs* were relevant primarily as potential barriers, and the *Implementation climate* and *Readiness for implementation* constructs were relevant primarily as facilitating factors. 

### 4.1. Influencing Factors Relevant as Barriers and Facilitators

Regarding patient needs and resources, we displayed that some of the DeCM interventions disregard the specific needs of PwD and caregivers and that the complexity of DeCM might increase the threshold for PwD and caregivers for using it. Ignoring these needs affects the effectiveness of implementation, while patient-centered initiatives can increase the success of implementation [13,25]. Especially in the context of dementia care, organizations with a high degree of patient-centeredness have been shown to have more efficient and positive implementation outcomes and change processes [26]. Closely related to these findings is the present skepticism about the relative advantage of DeCM. The perception that an intervention is advantageous in terms of its effectiveness or efficiency greatly increases the likelihood of implementation [13,25]. There are objections that specific DeCM interventions do not add value in relation to the status quo. We assume that this judgment is highly dependent on the characteristics of the reviewer. We repeatedly found contradictory evaluations and disagreements between evaluators across topics, which could be at least partly related to their profession. Understandably, people from the counseling setting see a great advantage for PwD and caregivers in detailed and time-consuming counseling, while GPs see time-consuming counseling as a hurdle due to the extreme overload in their practices. Our assumption that the evaluation of concrete measures is strongly dependent on the individual or job-related characteristics of the reviewers is also supported by the fact that of the top 5 CFIR constructs, three are simultaneously barriers and facilitators. From a meta-perspective, this result reveals an essential finding. Regarding the intersectoral care of PwD, care actors have diverse and at least partially conflicting ideas and needs regarding what PwD and caregivers need, whether and to what extent there is a desire for change, and to the extent to which very different care actors cooperate across sector boundaries. Our finding that the existence and quality of established network structures is a key influencing factor for implementation from the stakeholders’ perspective is also evident in other participatory studies. Here, too, it is concluded that efficient health care, especially in rural areas, depends crucially on cooperation and existing formal and informal networks between providers of health, social and administrative services [27]. 

Overall, our analysis identified discharge management as one of the main topics and a highly relevant barrier to implementing DeCM in SW. This is in line with other findings that indicate a high demand for evidence-based concepts for the follow-up care of PWD after an inpatient stay [28,29]. The DCM precisely aims to close this gap through its intersectoral approach. The results from current field studies in intersectoral care will soon provide important information on the effectiveness and efficiency of this approach [29]. However, our findings show that there is a strong perceived potential for DeCM to solve the current challenges in intersectoral care. Overall, there is a high need for DeCM. It is seen as contributing to overcoming the existing sectoring in the German health care system. According to the reviewers, dementia care managers are a key to this goal. Their use would represent a considerable advantage over current care: for PwD and caregivers, they would be a source of support, guidance, and assurance, and for GPs, they would relieve the burden by taking over tasks. We showed that their deployment would not create duplicate structures in the existing care system. In contrast, their services are seen as complementary. Overall, DeCM seems to meet the existing needs of PwDs and caregivers, especially through a holistic approach and comprehensive counseling and support services. 

### 4.2. Influencing Factors Relevant as Barriers

The results indicate that a high number of regional stakeholders in dementia care still need to be engaged. According to the results, this circumstance represents the greatest barrier on the path to implementing the adapted DeCM standard in SW. That the identification and involvement of relevant stakeholders in the early planning stages of intervention is crucial has also been shown by other studies on factors of successful implementation of health interventions [30]. This information is relevant in two respects. On the one hand, persons who are highly relevant for successful implementation were identified. The targeted identification and involvement of relevant stakeholders play a significant role. A team strategically composed of decision-makers, stakeholders, and advocates increases the prospect of implementation success [13]. In addition, the results show that the DeCM standard itself should be extended. Although the high number of dates coded in the engagement construct also results from the fact that the same stakeholders were repeatedly named across interviews, this possible limitation must be put into perspective. According to our understanding, the high degree of agreement between the reviewers from different sectors speaks precisely to the fact that these identified persons and institutions are *the* essential players in dementia care in SW. Therefore, it would be critical to integrate them into the implementation strategy or the adapted DeCM standard itself. Furthermore, our findings show that GPs might oppose DeCM and reject its implementation in SW, affecting trialability. Specifically, this skepticism relates to the changed competence range by deploying dementia care managers and the presumed increased workload for GPs. The literature suggests that time constraints related to the diagnosis and management of dementia are considered a barrier to the provision of dementia care by GPs [31,32]. This identified barrier may need to be put into perspective, as previous studies have shown that GPs accept and appreciate the original DCM [4] and benefit from integrated care and case management models [31]. Furthermore, GPs seem to hold a positive attitude toward it and consider DCM a relief in caring for PwD; additionally, they consider dementia care managers competent and appreciate the time spent with them [4]. Nevertheless, attention should be given to this aspect in the further course of implementation, as this estimation by the reviewers is based on their practical work. Therefore, for our implementation trial, we are planning a series of events and network meetings to familiarize GPs in SW with DeCM. 

### 4.3. Influencing Factors Relevant as Facilitators

We have found perceived fit between the adapted DeCM standard and existing health care networks, internal processes, and infrastructure in SW. Thus, according to our results, DeCM faces a positive implementation climate in SW, which is expected to facilitate its implementation in the local healthcare setting. What is remarkable about the results is the signaled interest and willingness of some of the interviewed reviewers (some being leading staff) to employ dementia care managers in their institutions. The finding that leading staff endorse DeCM gives reason for optimism in the light of other studies on the implementation success of dementia care interventions. For it has been demonstrated that positive attitudes and active leadership involvement in implementation were proven to be associated with implementation success [26,33]. The numerous local initiatives, alliances, and committed, highly connected key players are very important factors in the implementation process. This was already evident in the recruitment of reviewers. The co-researchers were very quickly able to recruit relevant interview partners from all relevant sectors and different hierarchical levels to be peer reviewers. During the interviews, we identified numerous other local actors, associations, individuals, and institutions with strong networks among each other. The existence of these networks and initiatives may be *the* facilitating factor for the implementation of DeCM in SW. However, our findings also show that not all stakeholders in SW are involved in these networks. Cross-sectoral cooperation often takes place between certain institutions or through committed individuals rather than through sophisticated and comprehensive collaborative local structures. One consequence of this result is that in the next few months, together with the relevant project partners, we will be tackling these critical interfaces and strengthening cooperation between relevant stakeholders to increase the likelihood of sustainable implementation.

### 4.4. Strengths and Limitations

Our design approach is in line with the requirements of qualitative research. For example, by considering aspects such as communicative validation, intersubjective traceability, or the fit of survey and evaluation methods through strict application of the CFIR framework and documentation in the form of a research diary, we aim for *trustworthiness* [34] (see also description in Table S1). Through purposive sampling, relevant stakeholders from the main health sectors could be identified. Additionally, our sample depicts both the hierarchical and gender-related characteristics of the care landscape. This enabled us to gain detailed and action-guiding insights into the inhibiting and beneficial circumstances for the implementation of the adapted DeCM in routine care. Through this case selection, we aim to achieve transferability [34] of the results to at least similar contexts in other regions. Further strength lies in the possibility of deriving direct action-guiding measures from the results. For example, modifications in the DeCM standard itself stem from the presented results. Additionally, the results were presented to the project partners involved in the study, where the results can be used as a basis for, e.g., preparing the implementation strategy, implementing public relations initiatives, or promoting future networking efforts. Since the results come from a participatory study, high practical relevance can be assumed.

Nevertheless, some study limitations can be identified. These limitations relate to the generalizability of the results, a possible sampling or selection bias, interviewer effects and characteristics, as well as the unsuccessful triangulation of the data. Due to the small number of interviewees, it cannot be assumed that the evaluators are representative of the population of all stakeholders in the region. All data collected refer exclusively to the SW region and can therefore not be generalized to all regions in Germany. Our results show that the implementation of DeCM, as a process and outcome, is highly dependent on regional particularities and on the kind and number of stakeholders involved. For that reason, we do not intend to draw general implications for the whole German region. In addition, the peer reviewers were selected by the co-researchers, which means that a certain degree of sampling bias and a selection bias in favor of reviewers open to DCM cannot be ruled out. Accordingly, there is also the possibility that we could not comprehensively assess the barriers. However, we cautiously assume that the data are saturated, as there are indications that saturation can be reached within 12 interviews [35]. From a methodological point of view, there are also limitations regarding the generation of data. Although the interviews conducted by the co-researchers allow a practical collection of information, the co-researchers are not experienced and trained interviewers. In addition, the observation protocols suggest significant differences in the way the interviews were conducted, which might have led to biased data. The guiding questions were not always used, the rating of the relevance of the statements was not carried out by all co-researchers, and the amount of data generated differed between the interviews. For this reason, we decided not to weigh the barriers in the process of data analysis. Interviewer effects cannot be ruled out either. There are different hierarchical relationships between co-researchers and peer reviewers that could have influenced the response behavior. In addition, the peer reviewers were aware that the co-researchers participated in the adaptation of the DCM standard. The direct summarizing protocol by the research interns could also be criticized. To a certain extent, they acted as “gatekeepers”. We paid great attention to communicative validation and the inclusion of ratings during the interview. This feedback loop served the quality assurance of the protocol and, therefore, the later analysis. Nevertheless, it cannot be ruled out that important information was not recorded or was recorded incorrectly. 

However, our study provides concrete indications of possible critical determinants of the future transfer of the evidence-based DCM standard into health care practice in Germany. Our approach to involve multiple stakeholders in a barrier analysis could serve as a guide for implementation efforts in other regions to follow. We have shown that the implementation process benefits from a comprehensive participatory approach. As academic researchers, we received strategic, site-specific information on possible barriers to the implementation strategy. Moreover, the participatory approach of the implementation study (*Participatory pilot study DelpHI-SW*) in general is seen as a facilitating factor by some reviewers. Accordingly, our findings support the literature on the importance of involving multiple stakeholders in the preparation and practical implementation of complex health intervention programs and indicate that “… when synergy generated positive outcomes …, those outcomes generated new synergy”. [36] (p. 334). This approach can have numerous benefits for health intervention and implementation research, e.g., an increased recruitment capacity, quality of outcomes, sustained programs after a study ends, and the generation of systemic changes [9,36,37]. As presented above, participatory approach allows the early identification and consideration of conflicting needs and demands of care providers on DCM. In this sense, we aim to continue the participatory approach in the future course of implementing the new DeCM standard in the region. Furthermore, we assume that the inclusion of existing regional networks at a very early stage of future implementation efforts on DCM will increase the prospect of success. This requires an early evaluation of which stakeholders are not or are not yet adequately integrated into local network structures. In particular, the participation of these target groups in implementation projects should be promoted. Through cooperation in the framework of implementation projects, new links between stakeholders can be created at this early stage, which can also be transferred to care practice and result in the sustainable improvement of intersectoral care. 

## 5. Conclusions

We demonstrated that the participatory approach leads to the development of a very targeted new DeCM standard that utilizes regional resources without creating new health care structures or processes. Furthermore, through the intersectoral involvement of further experts as reviewers and the theoretical foundation of the barrier analysis, critical elements are systematically identified already in the implementation planning process. The barrier analysis allowed us to generate comprehensive feedback and actionable findings to strengthen the implementation efforts. Overall, all of these aspects might increase the prospects of the feasibility, acceptance, and implementability of the DeCM standard in routine care. Investigating this is the mandate for subsequent studies. The application of the CFIR framework makes the results connectable to further planned evaluations at later phases of the study. Furthermore, the findings can provide guidance for the implementation of comparable interventions and the design of similar projects.

## Figures and Tables

**Table 1:** Summary of the key results, by barriers and facilitators.

Barrier	Facilitator
**CFIR constructs relevant as barriers and facilitators**	
**Patients needs and resources**	**Patients needs and ressources**
Disregards specific needs of PwD and care givers Disproportionality of specific DeCM interventions No low-threshold program for PwD and relatives	Dementia care manager as a support person Improved care of PwD in the context of discharge management Consideration of the specific needs of those affected Consideration of comprehensive counseling and support services Solving existing challenges in intersectoral care of PwD
**Relative advantage**	**Relative advantage**
Increased workload for GPs Lack of added value of specific DeCM interventions	Dementia care managers as centerpiece of DeCM Possibility of overcoming sectorization in the health care system Relieving the burden on GPs
**Cosmopolitanism**	**Cosmopolitanism**
Lack of communication and cooperation at sector boundaries Lack of telematic/digital infrastructure Hierarchical care structures	Participatory, intersectoral character of the study COVID 19-induced digitalization in all care sectors Existing regional network structures
**CFIR constructs relevant exclusively as barrier or facilitator**	
**Engaging**	**Implementation climate**
Lack of involvement of relevant stakeholders for the implementation process and within specific DeCM interventionsy	High perceived need for DeCM High willingness to implement DeCM Fit between DeCM and existing networks, internal processes, and infrastructure Deployment of dementia care managers is desired Existence of flexible and motivated stakeholders
**Trialability**	**Readiness for implementation**
Overloading of GP practices High implementation costs due to the high complexity of the DeCM standard and the large number of health care sectors involved Dependence on motivation, attitude and persuasion of the stakeholders involved	Interest in implementing DeCM Desire to hire dementia care managers Resources available for implementation

*CFIR* Consolidated Framework for Implementation Research; *DeCM* dementia care management; *GPs* General Practioners; *PwD* People with Dementia.

## Data Availability

Not applicable. For data protection reasons, no raw data can be made available, as it is highly likely that conclusions can be drawn about the individual peer-reviewers from this data.

## Supplementary Materials

The following supporting information can be downloaded at: https://www.mdpi.com/article/10.3390/ijerph19095478/s1, Table S1: Standards for Reporting Qualitative Research (SRQR) checklist; Table S2: Overall results of the coding process, separately by barriers and facilitators and assigned to CFIR domains and constructs; Supplementary Materials File S1: Interview guide; Supplementary Materials File S2: Oberserver Guide.
