# Supplementary material for: Adapting a Dementia Care Management Intervention for Regional Implementation: A Theory-Based Participatory Barrier Analysis
Source: Int J Environ Res Public Health. 2022 Apr 30;19(9):5478. doi: 10.3390/ijerph19095478 (PMC9101206; doi:10.3390/ijerph19095478)
Supplement: Supplementary file 1 [file ijerph-19-05478-s001.zip › Supplementary Materials File S1 Interview guide.pdf]

Supplementary Material III: Interview guide.

Question	Background/Subject
What do you find better/worse about the proposed approach compared to the current approach?	Perception of advantages of the adapted dementia care management (DeCM) standard compared to "care as usual" → **Relative advantage**
It is possible that the new care model will have to be adapted once again. Are there components that should be retained in any case?<br>Which elements should be adapted? → Why?	The extent to which DeCM components presented need to be adapted, tailored, refined, or redesigned to meet local requirements → **Adaptability**
How complex do you consider the implementation of the new care model to be? Rating!!<br>• Expenditure of time<br>• Workload<br>• Extent of changes that must be done for the implementation<br>• Communication between departments/ care sectors<br>At which points/where in the process of care do you see complications in putting DeCM into practice?	Perceived difficulty of implementing DeCM in health care practice → **Complexity**
Do you think that the new approach fits the needs of the people treated by us (people with dementia, caregivers)? If not, why? Which kind of difficulties could appear for them during implementation of the new care model?	The extent to which patients' needs and the barriers and facilitators to addressing those needs are accurately known
When you think about your organization: e.g., size, equipment, social atmosphere, progressiveness, premises, organization's working procedures, etc.<br>(a) Which circumstances could hinder the implementation of DeCM in practice?<br>(b) Which circumstances could support the implementation?	Structural characteristics of the involved organizations
How do you think the corporate culture/guidelines will affect the implementation of the new DeCM? Does your corporate culture fit with the adapted DeCM?	Describes values, norms, and fundamental assumptions in an organization and how they (could) influence the implementation of DeCM → **Corporate culture**
Do you think there is a need for this new form of dementia care?	The extent to which those involved perceive the current situation as intolerable → **Need for change**

Do you consider the resources of your organization to be sufficient to implement DeCM (e.g., finances, qualification of staff, time, premises)?<br>Who needs to be involved so that DeCM can be implemented in our region?<br>Who are the people/organizations that can drive the successful implementation of DeCM?	Are the provided resources sufficient for the realization and current operation?<br>→ **Available resources**<br>→ **Process/Integration**
Overall, do you think the adapted DeCM standard can work in your field? If not: why not; what stands in the way of implementation?	→ **Attitude towards DeCM**
I provided some examples of the adapted DeCM standard. Do you have any ideas on how we can improve care for PWDs and their families in the region?<br>Do you miss any important points?<br>Have we ignored important barriers/circumstances during adaption?	Capture information that has not yet been considered during development.<br>**Gather more info on resources or barriers in the area.**
