# Supplementary material for: Adapting a Dementia Care Management Intervention for Regional Implementation: A Theory-Based Participatory Barrier Analysis
Source: Int J Environ Res Public Health. 2022 Apr 30;19(9):5478. doi: 10.3390/ijerph19095478 (PMC9101206; doi:10.3390/ijerph19095478)
Supplement: Supplementary file 1 [file ijerph-19-05478-s001.zip › Supplementary Materials File S2 Observer guide.pdf]

Supplementary Material IV: Observer guide.

Date/Time

Health care sector

Name of the Reviewer/Interviewer

Characteristics of the interview (e.g., during working hours, place, recruitment etc.)

General remarks on the interview (e.g., process, disturbances, according to plan etc.)

Specifics during the interview (Were there any problems in the realization of the planned procedure? What was done differently and why? Were there any open questions that could not be resolved?)
